# Effects of Pleistocene sea-level fluctuations on mangrove population dynamics: a lesson from *Sonneratia alba*

**DOI:** 10.1186/s12862-016-0849-z

**Published:** 2017-01-18

**Authors:** Yuchen Yang, Jianfang Li, Shuhuan Yang, Xinnian Li, Lu Fang, Cairong Zhong, Norman C. Duke, Renchao Zhou, Suhua Shi

**Affiliations:** 1 State Key Laboratory of Biocontrol and Guangdong Provincial Key Laboratory of Plant Resources, Sun Yat-sen University, Guangzhou, 510275 China; 2 Hainan Dongzhai Harbor National Nature Reserve, Haikou, 571129 China; 3 Trop WATER, James Cook University, Townsville, Quennsland Australia

**Keywords:** Gene flow, Genetic diversity, Indo-West Pacific, Mangroves, Pleistocene glaciations

## Abstract

**Background:**

A large-scale systematical investigation of the influence of Pleistocene climate oscillation on mangrove population dynamics could enrich our knowledge about the evolutionary history during times of historical climate change, which in turn may provide important information for their conservation.

**Results:**

In this study, phylogeography of a mangrove tree *Sonneratia alba* was studied by sequencing three chloroplast fragments and seven nuclear genes. A low level of genetic diversity at the population level was detected across its range, especially at the range margins, which was mainly attributed to the steep sea-level drop and associated climate fluctuations during the Pleistocene glacial periods. Extremely small effective population size (Ne) was inferred in populations from both eastern and western Malay Peninsula (44 and 396, respectively), mirroring the fragility of mangrove plants and their paucity of robustness against future climate perturbations and human activity. Two major genetic lineages of high divergence were identified in the two mangrove biodiversity centres: the Indo-Malesia and Australasia regions. The estimated splitting time between these two lineages was 3.153 million year ago (MYA), suggesting a role for pre-Pleistocene events in shaping the major diversity patterns of mangrove species. Within the Indo-Malesia region, a subdivision was implicated between the South China Sea (SCS) and the remaining area with a divergence time of 1.874 MYA, corresponding to glacial vicariance when the emerged Sunda Shelf halted genetic exchange between the western and eastern coasts of the Malay Peninsula during Pleistocene sea-level drops. Notably, genetic admixture was observed in populations at the boundary regions, especially in the two populations near the Malacca Strait, indicating secondary contact between divergent lineages during interglacial periods. These interregional genetic exchanges provided ample opportunity for the re-use of standing genetic variation, which could facilitate mangrove establishment and adaptation in new habitats, especially in the context of global climate changes.

**Conclusion:**

Phylogeogrpahic analysis in this study reveal that Pleistocene sea-level fluctuations had profound influence on population differentiation of the mangrove tree *S. alba*. Our study highlights the fragility of mangrove plants and offers a guide for the conservation of coastal mangrove communities experiencing ongoing changes in sea-level.

**Electronic supplementary material:**

The online version of this article (doi:10.1186/s12862-016-0849-z) contains supplementary material, which is available to authorized users.

## Background

Mangrove plants constitute a highly productive ecosystem of both ecological and economic importance that lies at the interface of terrestrial and marine environments [1]. They provide essential support to a variety of terrestrial and marine species through nutrient and organic matter sinking and transformation, protection from coastal erosion, and sediment control [2–4]. Mangroves also play crucial roles in daily human life, by creating fisheries, supplying primary raw materials for chemical and medical industries, and protecting against floods and tsunamis, especially for those living within 10 km of an area harboring significant mangrove forests [3, 5–7]. However, mangrove communities have faced rapid decline; between 35 and 86% of the area once home to mangroves has been lost over the past 30 years, due to both climate change and human activity [8]. This loss poses a serious ecological problem and requires urgent conservation in the face of further increases in climate fluctuation. To better protect mangrove communities, a comprehensive understanding of the evolutionary demography of mangrove plants during historical climate oscillations is necessary.

Over the last 3 million years, Earth has experienced multiple glaciation events associated with steep sea-level fluctuations and climate changes that are thought to have shaped the modern biogeography of both terrestrial and marine organisms [9–11]. Unlike terrestrial plants, most mangrove species have water-dispersed seedlings that can float for an extended period of time [4], which may play a role in the geographical distribution and population dynamics of mangrove plants. However, traditional views have proposed that large landmasses could halt propogule dispersal, especially during glaciation, resulting in the genetic structure within mangrove species [12–14]. Many phylogeographical studies have linked genetic divergence with glacial vicariance in many mangrove species across the Indo-West-Pacific (IWP) region [15–24].

During interglacial periods of the Pleistocene, the re-submergence of land shelves was thought to provide corridors for genetic exchanges between the oceanic regions isolated during glaciation [25]. Using molecular markers, several studies have provided evidence supporting the hypothesis that mangroves can detour around vast landmasses or across open seas (oceans) and can effectively colonize geographically distant regions [22, 26–28]. However, in the case of *Rhizophora mangle* [29] and *R. mucronata* [23], ocean circulations, similar to land barriers, were proposed to play an important role in preventing gene flow and maintaining high genetic divergence. It has been suggested that sea-drift long-distance dispersal (LDD) is less effective than previously thought [12]. Thus, whether substantial genetic exchanges exist among different oceanic regions during the Pleistocene interglacial periods remains elusive and requires further confirmation.

*Sonneratia alba*, a widespread mangrove species in the IWP region, is distributed from East Africa through Southeast Asia to southern Japan and northeastern Australia. Unlike species in the Rhizophoraceae family, which have viviparous propagules of 15 to 70 cm long, *Sonneratia* species produce small irregular seeds of 7 to 12 mm long [4, 30]. Mature fruits of *Sonneratia* species, which are characterized by a separated calyx with exposed seeds, can float on sea currents and be carried some distance by sea currents before seed release [4]. Thus, the dispersal ability of *Sonneratia* species may be different from that of *Rhizophora* species. A previous population genetic study of *S. alba* identified 12 highly divergent genes and 59 undifferentiated genes between two nearby populations in Hainan, China [31], suggesting that inter-population gene flow led to homogeneity in most genes, whereas local adaptation overcame the homogenization role of gene flow in a minority of genes. Another study on a congeneric species *S. ovata* showed that drops in the Pleistocene sea level might lead to the loss of polymorphism in several populations from the South China Sea (SCS) [32]. These findings have enriched our knowledge of genetic diversity of *Sonneratia* species. However, these studies were conducted over a relatively small area, and a more comprehensive phylogeoraphical study covering the entire range is required for *Sonneratia* species.

In this study, we sequenced three chloroplast fragments and seven nuclear loci for 22 populations of *S. alba* across its range (Table 1) to assess the influence of fluctuations in Pleistocene sea level and climate on patterns of genetic diversity in mangrove species. Furthermore, we tested whether there was substantial genetic exchange between different oceanic regions. We wish to provide new insights into the evolutionary history of mangrove plants exposed to Pleistocene sea-level fluctuations.

## Results

### Genetic diversity of *S. alba* at the population and species levels

For the chloroplast locus, the length of the three concatenated fragments was 4287 bp for each of the 215 individuals from 22 populations of *S. alba*. Eight single nucleotide polymorphisms (SNPs) were identified in the chloroplast locus and produced seven chlorotypes. Only three populations, namely, those from Kuta, Indonesia (IKT), Cebu, Philippines (PCB) and Morotai, Indonesia (IBM), exhibited polymorphism. The nucleotide diversity (θ_π_) was 0.140, 0.050 and 0.140 per kb (/kb), respectively, while the nucleotide polymorphism (θ_W_) was 0.250, 0.080 and 0.170/kb, respectively (Additional file 1).

**Fig. 1:**
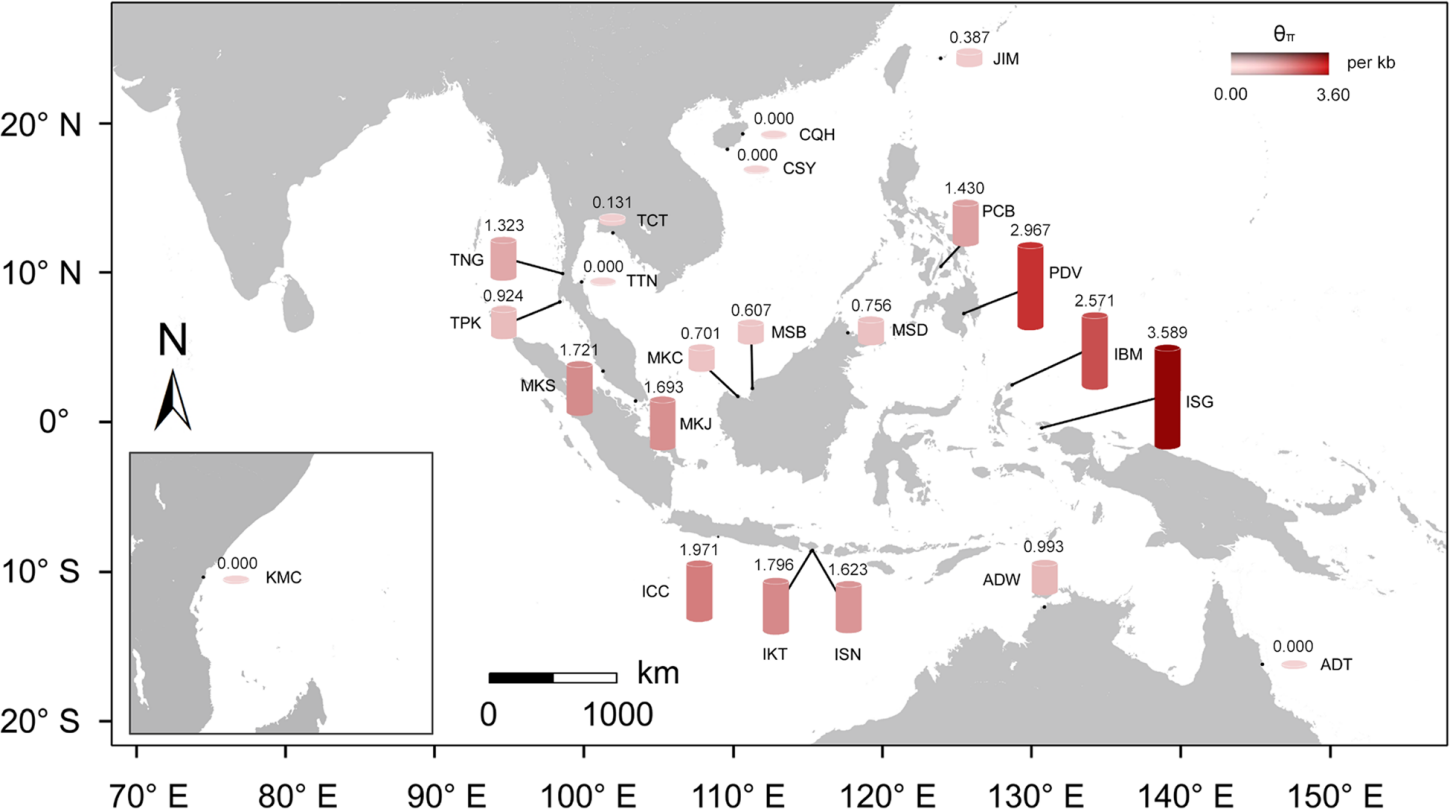
A heatmap of nucleotide diversity (θ_π_) of 22 populations of *Sonneratia alba*. The color depth and the height of the cylinder are proportional to the level of θ_π_. Population abbreviations were defined in Table 1

The length of the seven nuclear loci ranged from 609 to 1382 bp. At these seven loci, between 11 and 36 SNPs were identified with θ_π_ ranging from 1.550 to 4.920/kb and θ_W_ ranging from 2.170 to 3.650/kb (Additional file 1). At the population level, *S. alba* hosted a relatively low level of genetic diversity (Fig. 1; Additional file 2). Populations at the range margins exhibited no or extremely low genetic diversity; for example, no polymorphism was detected in the populations from China (CQH and CSY), Kenya (KMC) and northeastern Australia (ADT). By contrast, geographically central populations harbored relatively high genetic diversity (Fig. 1; Additional file 2). The populations from Davao, Philippines (PDV; the means of θ_π_ and θ_W_ were 2.967 and 2.549/kb, respectively) and West Papua, Indonesia (ISG; the means of θ_π_ and θ_W_ were 3.589 and 1.913/kb, respectively) had the highest and second highest diversity, respectively (Additional file 1). Populations located at the Indo-Pacific boundary (including MKJ, MKS, IKT and ISN) also exhibited high genetic diversity.

### Genetic differentiation among populations

**Fig. 2:**
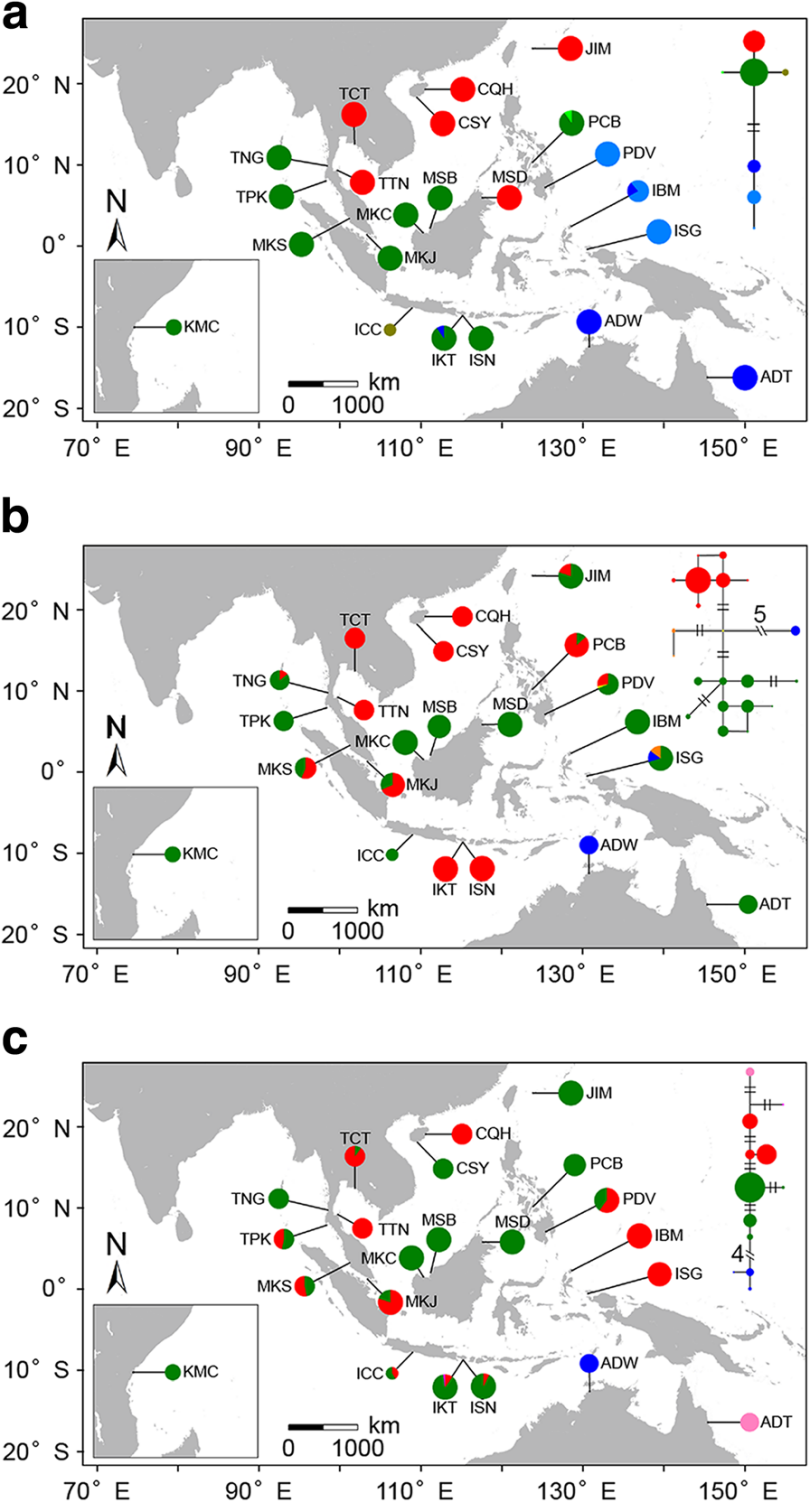
Geographic distribution of haplotypes and Median-Joining network for the chloroplast locus (**a**) and two nuclear loci, rpl9 (**b**) and cpi (**c**), in 22 populations of Sonneratia alba. Each haplotype was represented by one single circle and haplotype frequency was illustrated by circle size. Haplotypes with close relationship were denoted by the same color. The number of mutations is 1 unless otherwise indicated. Population abbreviations were defined in Table 1

Haplotype networks of both chloroplast and nuclear loci showed strong genetic differentiation among different oceanic areas in the IWP region (Fig. 2; Additional file 3). The seven chlorotypes fell into two major clades separated by two mutational steps; one of which was restricted to the populations from the SCS and Indian Ocean, as well as those from Iriomote, Japan (JIM), Sandakan, Malaysia (MSD) and Cebu, Philippines (PCB), while the other one was mainly found in the West Pacific populations, including those from Davao, Philippines (PDV), West Papua and Morotai, Indonesia (ISG and IBM) and Australia (ADW and ADT, Fig. 2a). The geographical distributions of the two clades correspond to the Indo-Malesia and Australasia regions. Within each clade, there was further subdivision separated by one mutational step between the SCS and the Indian Ocean populations and between the Australian (ADW and ADT) and Philippine and West Indonesian (PDV, IBM and ISG) populations.

A similar but more complex genetic structure was observed at the seven nuclear loci (Fig. 2). Consistent with the cpDNA data, the strongest differentiation was found between the populations from the Indo-Malesia and Australasia regions at five of the seven loci (*rpl9*, *cpi*, *ppi*, *cci* and *nhx2*; Fig. 2b, and c; Additional file 3a, c and e). Within the Indo-Malesia region, the genetic break between the SCS and the Indian Ocean populations was recovered for all but one locus (*phi*), which was nearly monomorphic in this region (Fig. 2; Additional file 3). The haplotypes of these two regions were separated by two to 18 mutational steps. With regard to the Australasia region, the haplotypes of the eastern Australia population (ADT) were closely allied with those from the Indian Ocean populations at two of the seven loci (*rpl9* and *ppi*), while at the *cpi* and *idr* loci, the ADT population exhibited a closer relationship to the populations from the SCS.

**Fig. 3:**
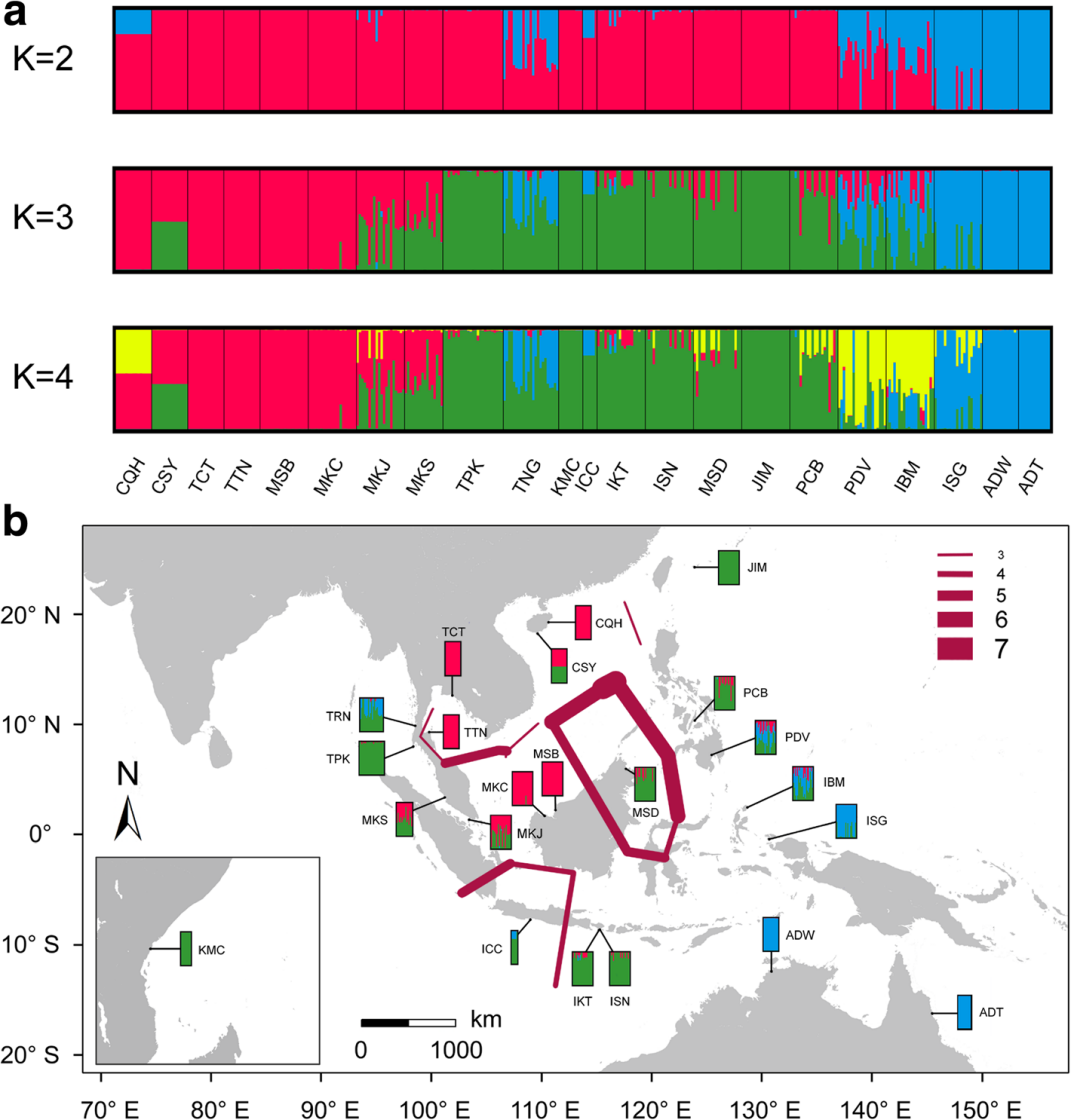
Likely genetic clusters and geographical barriers existing among 22 populations of *Sonneratia. alba*. **a** Bayesian clustering analysis of the seven nuclear genes for 22 populations of *S. alba* using STRUCTURE. To show the hierarchical population structure across the IWP region, in addition to the optimal clustering of K =3, the clusterings under K = 2 and K = 4 were also given. **b** Putative geographical barriers identified within the IWP regions. The red line represents detected barriers and the thickness corresponds to the number of genes that support this barrier. The bars showed the result of Bayesian clustering analysis when K = 3. Population abbreviations were defined in Table 1

Bayesian clustering analysis presented a clearer view of the genetic structure of *S. alba*. Under the optimal clustering of K = 3 (Additional file 4), the 22 populations fell into three clusters, in concordance with our cpDNA analysis (Fig. 3a). The populations from the SCS region, including those from China (CQH and CSY) and the eastern coasts of Malay Peninsula (TCT, TTN, MSB and MKC), formed one cluster (we denoted it as “the SCS cluster”), while those from the Indian Ocean (TPK, TNG, KMC, ICC, IKT and ISN) as well as Iriomote, Japan (JIM), Sandakan, Malaysia (MSD) and Cebu, Philippines (PCB), formed the second cluster (we denoted it as “the Indian Ocean cluster” because most populations in this cluster are from the coasts of the Indian Ocean). Populations from New Guinea (ISG) and Australia (ADW and ADT) formed the last cluster. When K = 2, there was a clear split between the Indo-Malesia region and the Australasia region. The two clusters from the SCS and the Indian Ocean were merged into one cluster, while the populations from New Guinea and Australia formed the other. The geography-associated genetic clustering of *S. alba* is also supported by the NJ tree (Additional file 5).

Using the Barrier software, putative geographical barriers were identified among different oceanic regions across the IWP region (Fig. 3b). The strongest barriers existed around the Sandakan population (MSD), which corresponds to the coastal line of the Sulu Sea. Two other barriers were identified between the SCS and the Indian Ocean populations, as well as between the SCS and the Java Sea populations, which could result in the genetic breaks between the SCS and other regions.

**Fig. 4:**
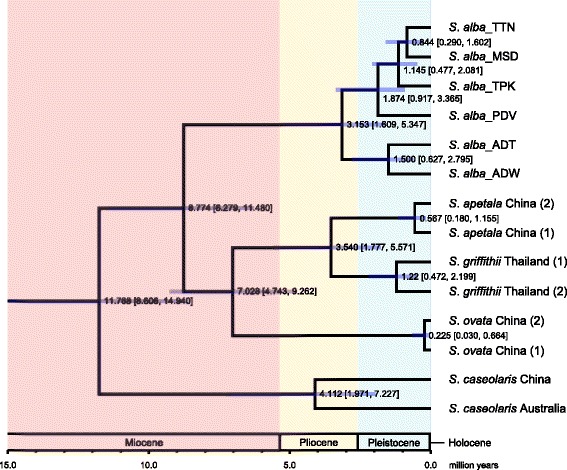
Divergent times of different lineages of *Sonneratia alba* and among five *Sonneratia* species. The scale bar is 1.0 million years (MYA). The value and purple bar at each node indicate the estimated divergent time (MYA) with a 95% of the highest posterior density (HPD) interval, respectively

The divergence times between the major genetic lineages of *S. alba* were estimated using the BEAST software. The deepest divergence was observed between the lineages from the Indo-Malesia and Australasia regions and was estimated to be 3.153 million years ago (MYA; 95% of the highest posterior density (HPD): 1.609–5.347 MYA, Fig. 4). Within the Indo-Malesia region, the SCS lineage (TTN) was estimated to diverge from the Indian Ocean lineage (TPK) 1.145 MYA (95% HPD: 0.477–2.081 MYA). The splitting time between the populations from the Sulu Sea (MSD) and the SCS (TTN) was 0.844 MYA (95% HPD: 0.290–1.602 MYA). The Davao population from the Philippines (PDV) was the earliest to diverge from other populations in the Indo-Malesia region (1.874 MYA; 95% HPD: 0.917–3.365 MYA). Within the Australasia region, the populations from eastern and northwestern Australia began to diverge 1.500 MYA (95% HPD: 0.627–2.795 MYA).

### Genetic admixture at the Indo-Pacific boundary

At five of the seven loci (*rpl9*, *cpi*, *ppi*, *idr* and *nhx2*), there was haplotype mixing between the SCS and the Indian Ocean lineages in the two populations near the Malacca Strait (MKJ and MKS, Fig. 2b, c, Additional file 3a, d and e). Bayesian clustering analysis also revealed apparent genetic admixture in these two populations (Fig. 3a). When K = 3, approximately 59.0 and 48.8% of genetic composition of MKJ and MKS populations, respectively, was derived from the SCS lineage, while 40.2 and 50.9% was derived from the Indian Ocean lineage. Similar genetic admixture was observed for other populations from Bali, Indonesia (IKT and ISN), Davao, Philippines (PDV) and Morotai, Indonesia (IBM), suggesting that secondary contact may have occurred at the boundary areas of different oceanic regions, especially at the Indo-Pacific boundary.

More interestingly, our results showed that two Chinese populations (CQH and CSY) were highly divergent and fell into different clusters at two loci: *cpi* and *nhx2* (Fig. 2c Additional file 3e). The CQH population shared the same alleles with the SCS lineage, whereas the CSY population was closely allied with the Indian Ocean lineage. Bayesian clustering analysis indicated that 51.6% genetic composition of the CSY population was derived from the Indian Ocean lineage, while the rest belonged to the SCS lineage (K = 3). In the Japanese population (JIM), haplotypes at four loci (*cpi*, *ppi*, *idr* and *nhx2*) hosted similar genetic composition with the Indian Ocean lineage (Fig. 2c, Additional file 3a, d and e), while two loci (*phi* and *cci*) fell into a cluster with the SCS lineage (Additional file 3b, c). Interestingly, this population exhibited an admixture of the two genetic lineages at the remaining locus *rpl9*. The mosaic distribution of the haplotypes of different nuclear loci suggested long-distance gene flow from the Indian Ocean to the SCS populations.

### Demographic history inference under the isolation with migration (IM) model

**Fig. 5:**
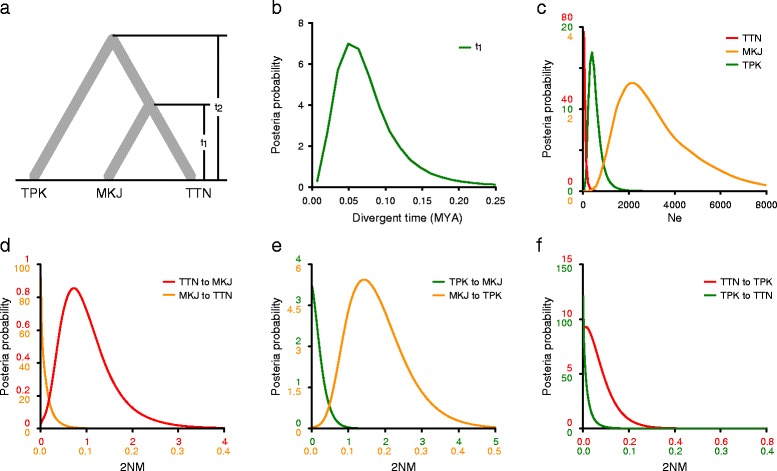
Probability density plots of the demographic parameters estimated using the isolation with migration model for the populations from the SCS (TTN), the Indian Ocean (TPK) and the Indo-Pacific boundary region (MKJ). **a** A schematic of isolation with migration model. Population abbreviations were defined in Table 2. **b** Probability density estimation of the formation time of the population MKJ (t_1_). **c** Probability density estimation of the effectively population sizes (Ne) of the three populations. **d**-**f** Probability density estimation of the migration rates between populations TTN and MKJ, TPK and MKJ and TTN and TPK, respectively

Simulations under the IM model provided strong evidence for inter-regional gene flow between the SCS and the Indian Ocean lineages via the Malacca Strait (Fig. 5). The three repeated runs produced unambiguous marginal posterior probability distributions for all population parameters and the smallest effective sample size (ESS) approached 70,000, suggesting a reliable estimation with a well-mixed chain. Because we did not obtain a good posterior distribution for divergence time between the SCS and the Indian Ocean populations (t_2_) by IMa2 analysis, the BEAST software was used to estimate the splitting time between these two oceanic regions (Fig. 4). The divergence time between the MKJ and the SCS populations (t_1_) was estimated to be 0.070 (95% HPD: 0.000–9.650), which corresponded to 0.049 MYA (95% HPD: 0.000–6.791 MYA) under the assumed generation time of 20 years and an estimated nucleotide substitution rate of 1.616 * 10^−9^ s/s/y by BEAST (Fig. 5b; Table 2). Notably, the effective migrations from the SCS to the Indian Ocean populations (2NM _TTN -> MKJ_ and 2NM _MKJ -> TPK_) via the Malacca Strait were significantly greater than zero (Fig. 5d and e; Table 2). The migration rate was estimated at 0.727 (95% HPD: 0.089–2.229; LLR = 7.259; *P* < 0.01) from the TTN population in the SCS to the MKJ population at the Malacca Strait, while that from the MKJ population to the TPK population in the Indian Ocean was 0.142 (95% HPD: 0.043–0.347; LLR = 9.344; *P* < 0.01). By contrast, the migration rate in the opposite direction was close to zero in both cases. These results suggest that secondary contact and asymmetric gene flow between the SCS and the Indian Ocean populations took place at the boundary region. The effective population sizes (Ne) of the TTN and TPK populations were estimated to be 44 (95% HPD: 0–748) and 396 (95% HPD: 132–1,451), respectively, while it was estimated to be 2,155 (95% HPD: 660–6993) for the MKJ population (Fig. 5c; Table 2).

**Table 1:** Locations and sample sizes of 22 populations of *Sonneratia alba* used in this study

Code	Locations	Longitude	Latitude	Sample size
CQH	Wenchang, Hainan, China	110°37’ E	19°13’ N	15
CSY	Yalong Bay, Sanya, Hainan, China	109°36’ E	18°13’ N	15
JIM	Nakama River, Iriomote, Japan	123°52’ E	24°17’ N	20
TCT	Klong Ta-kian, Chanthaburi, Thailand	101°54’ E	12°34’ N	15
TTN	Thong Nian Bay, Nakhon Si Thanmarat, Thailand	99°48’ E	9 °18’ N	15
TPK	Phuket, Thailand	98°23’ E	7°59’ N	25
TNG	Ngao, Ranong, Thailand	98°32’ E	9°52’ N	23
MSB	Sibu, Salawa, Malaysia	111°14’ E	2°10’ N	20
MKC	Kuching, Salawa, Malaysia	110°20’ E	1°37’ N	20
MKJ	Kukup, Johor, Malaysia	103°26’ E	1°20’ N	20
MKS	Kuala Lumpur, Kuala Selangor, Malaysia	101°15’ E	3°20’ N	16
MSD	Sandakan, Sabah, Malaysia	117°39’ E	5°55’ N	20
PCB	Cebu, Philippines	123°52’ E	10°18’ N	20
PDV	Davao, Philippines	125°25’ E	7°12’ N	20
IBM	Berebere, Morotai, Indonesia	128°40’ E	2°24’ N	20
ICC	Cilacap, Java, Indonesia	108°59’ E	7°44’ S	6
IKT	Kuta, Bali, Indonesia	115°10’ E	8°43’ S	20
ISN	Sanur, Bali, Indonesia	115°16’ E	8°41’ S	20
ISG	Sawinggwai, Gam Island, West Papua, Indonesia	130°37’ E	0°27’ S	20
ADW	Darwin, Australia	130°50’ E	12°28’ S	15
ADT	Daintree River, Australia	145°27’ E	16°17’ S	13
KMC	Mida creek, Kenya	39°58’ E	3°22’ S	10

## Discussion

### Low level of genetic diversity at the population level in *S. alba*

Our results show that, although *S. alba* hosts a relatively high level of genetic diversity at the species level, its genetic diversity at the population level was relatively low across its entire range, especially at the range margins (Fig. 1; Additional files 1 and 2). No polymorphism was detected within the populations from China (CQH and CSY, see also Zhou et al. [31]), northeastern Australia (ADT) and Kenya (KMC). Correspondingly, IM model-based demography history inference suggested that the Ne of the populations from TTN and TPK was 44 and 396, respectively, under the estimated substitution rate 1.616 * 10^−9^ s/s/y by BEAST (Fig. 5c; Table 2). Similar paucity of intra-species genetic diversity was also observed in another mangrove species, *Bruguiera gymnorrhiza* [33].

The paucity of genetic diversity at the population level could be attributed to repeated extinction-recolonization events induced by Pleistocene sea-level fluctuations. During glacial stages, mangrove plants in the IWP region suffered steep declines in suitable habitats due to drops in sea level and accompanying declines in temperature and humidity, which confined mangrove plants to few small refugia near the equator [9, 34]. Paleopalynology studies have identified continued but decreased pollen sediment from mangrove plants in the continental slope of the SCS during glacial periods, especially in the north SCS [35, 36]. This result suggests that during glaciations, mangrove populations experienced steep bottlenecks and even extinction, leading to a considerable loss of genetic diversity at the population level, especially at the range margins. Moreover, during interglacial periods, the founder effect during recolonization led to a further decrease in the genetic diversity of peripheral populations, as suggested in other mangrove species such as *Avicennia marina* [37, 38] and *Hibiscus tiliaceus* [21], and in coral reefs [39]. Although local diversity may occasionally be inflated by genetic admixture from other differentiated populations (Fig. 5c; Table 2), the increase of Ne was restricted to few populations at the boundary region.

The impacts of global climate changes on a species mostly involve the loss of genetic diversity at the intraspecific level [40], while the level of genetic diversity is thought to reflect the plasticity of adaptation to changing environments [41–43]. Loss of genetic polymorphism and the potential for environmentally responsive plasticity within local populations can result in a reduction in the capacity to cope with global climate changes [44, 45].

### Genetic differentiation of *S. alba* among different oceanic regions

Across the IWP region, we identified strong genetic structure associated with geography in *S. alba*. The strongest divergence existed between the two centres of mangrove biodiversity: Indo-Malesia and Australasia (Fig. 3a; Additional file 5). A similar genetic break between these two areas has also been observed in other mangrove species, including *B. gymnorrhiza* [33] and *R. stylosa* [24]. Furthermore, the species composition of mangroves in the Indo-Malesia region is distinct from the Australasia region. Duke et al. [14] reported that 11 of the 58 species distributed in the IWP region are restricted to the Indo-Malesia region, while between seven and nine species are exclusively distributed in the Australasia region. The splitting time between populations of *S. alba* of these two regions dates back to 3.153 MYA (95% HPD: 1.609–5.347 MYA, Fig. 4), corresponding to the Late Pliocene period (2.58–3.60 MYA). This result indicates that the divergence of mangrove plants between Indo-Malesia and Australasia took place prior to the Pleistocene glaciations. Since the Eocene to Early Pliocene (from approximately 56.0 to 5.0 MYA), the geography of the Indo-Australian Archipelago (IAA, from Malay Peninsula through the islands of Indonesia and Philippines to New Guinea) were dramatically shaped by the plate motions [46], which was thought to give rise to the evolutionary distinction between the Indo-Malesia and Australasia regions [39].

Our results revealed strong genetic differentiation within the Indo-Malesia region between the populations from the SCS and the Indian Ocean, but no apparent structure within either region. Haplotype networks of chloroplast and nuclear loci, as well as Bayesian clustering and phylogenetic analysis, revealed that two highly divergent genetic clusters separately dominated in the SCS and the Indian Ocean (Figs. 2 and 3a; Additional file 3 and 5). The dichotomous genetic break between the populations of these two areas has also been observed in other mangroves [15–17, 19, 24, 27, 33] and in marine animals [47–53]. The divergence time between the two lineages was estimated to be 1.874 MYA (95% HPD: 0.917–3.365 MYA, Fig. 4), corresponding to the early Pleistocene period. Genetic differentiation in marine organisms between the SCS and the Indian Ocean has been attributed to the repeated Pleistocene glaciations. The Malacca Strait, which is only 25 m deep, plays an important role in connecting the SCS and the Indian Ocean. Steep drops in the glacial sea level, as much as 116 m, closed the Malacca Strait and connected the Malay Peninsula to the three Sunda islands (Sumatra, Borneo and Java), which formed a physical barrier between the SCS and the Indian Ocean [54, 55]. This barrier cut off interregional seawater exchange, and thereby halted sea-drifted gene flow between the two areas. Glacial isolation could also explain the genetic differentiation of the Sandakan population (MSD) from the SCS populations (Fig. 3b). Sandakan is located in the southwestern coast of the Sulu Sea, which is bounded by numerous islands and shallow seas. During glacial periods, these islands and the exposed basins of shallow seas isolated this area from external oceanic regions, including the SCS and the Celebes Sea [54].

### Genetic admixture in *S. alba* at the Indo-Pacific boundary

Mangrove plants are characterized by sea-drifted propagules, which can facilitate LDD and are thought to play an important role in the modern distribution and genetic composition of mangrove species [4, 12, 14]. However, as described in the Introduction, due to geographical barriers, whether substantial genetic exchanges occur between different oceanic regions during the Pleistocene interglacial periods remains in doubt. Moreover, genetic admixture is difficult to distinguish from incomplete lineage sorting, which makes the investigation of inter-regional gene flow ambiguous for mangrove plants [56]. In this study, we provided strong evidence for secondary contact between the SCS and Indian Ocean lineages of *S. alba* at the Indo-Pacific boundary.

As shown in the haplotype networks, we identified a mixing of haplotypes from the SCS and the Indian Ocean lineages in two populations at the Malacca Strait (MKJ and MKS) without median haplotypes between the two highly divergent haplotype groups (Fig. 2b, and c; Additional file 3a, d and e). Bayesian clustering analysis suggests that approximately 59.0 and 48.8% of the genetic composition of these two populations belongs to the SCS cluster, while 40.2 and 50.9% came from the Indian Ocean cluster (Fig. 3a). These results suggest that secondary contact occurred at the Indo-Pacific boundary. Moreover, at most loci (*rpl9*, *cpi*, *ppi*, *cci* and *nhx2*), the two populations from the western coast of Thailand (TPK and TNG) hosted haplotypes from the SCS cluster, however, at *cpi*, one haplotype of the Indian Ocean cluster was identified in the Chanthaburi population from eastern Thailand (TCT, Fig. 2; Additional file 3), suggesting an asymmetric genetic exchange between the two regions. The results of demographic history inference under the IM model also revealed that the migration rates from TTN to MKJ and from MKJ to TPK were significantly greater than zero, but not in the opposite direction. This suggests that there was substantial and asymmetric gene flow from the SCS population via the Malacca Strait to the Indian Ocean population.

Liao et al. [28] attributed the similar genetic composition of *Ceriops tagal* populations on the two sides of Malay Peninsula to both historical gene flow via the leak of Kra Isthmus prior to the emergence of the isthmus (approximately 5.0 MYA) and the infrequent LDD detouring around the Malacca Strait. Such minor dispersal events may have a significant influence on the population dynamics of mangrove plants. In this study, the splitting time between the populations of *S. alba* from the SCS and the Indian Ocean was estimated at 1.145 MYA (95% HPD: 0.477–2.081 MYA, Fig. 4), while the divergence time of the population MKJ was 0.049 MYA (95% HPD: 0.000–6.791 MYA) under the IM model (Fig. 5; Table 2), both of which are quite recent and do not extend to the pre-isthmus period. Thus, secondary contact at the Indo-Pacific boundary in *S. alba* must have occurred during the Pleistocene rather than pre-Pleistocene era. During interglacial periods, the rise in sea level re-opened the Malacca Strait [55, 57], providing opportunities for genetic exchange in mangroves between the SCS and the Indian Ocean. Furthermore, between the two areas, the direction of surface ocean currents was highly affected by both the surface gradient of the sea level and seasonally alternate East Asian monsoon. Due to the low sea level in the Andaman Sea, currents extending from the SCS to the Indian Ocean are stronger and more dominant than those in the opposite direction [58]. Taking seasonal East Asia monsoons into account, surface sea water is driven from the SCS into the Indian Ocean in the winter, whereas it is carried from the Indian Ocean to the SCS in the summer [59]. Thus, sea currents via the Malacca Strait are mainly directed towards the Indian Ocean, providing a plausible explanation for asymmetric gene flow from the SCS to the Indian Ocean.

In contrast to Wee et al. [24], who proposed a blocking role of bifurcating ocean currents in preventing genetic exchange and maintaining genetic divergence between these two areas for *R. mucronata*, our results demonstrated that the Malacca Strait could provide opportunities for secondary contact during interglacial periods. For most mangrove species such as *Rhizophora*, mature propagules are available only in summer [4], which is misaligned with the major surface current from the SCS to the Indian Ocean in winter. By contrast, *S. alba* flowers and sets fruit throughout the year [4], highly increasing the possibility of sea-drifted LDD of *S. alba*. Differences in biological characteristics may contribute to the different extent of interregional genetic exchanges between *S. alba* and other mangrove species.

### Local adaptation due to standing genetic variation

One interesting finding of our study is that two Hainan populations (CQH and CSY) differ in haplotype clustering at two of the seven loci (*cpi* and *nhx2*, Fig. 2c; Additional file 3e). At the two loci, the Qionghai population shared a haplotype with the SCS lineage, while the haplotypes of the Sanya population clustered with the Indian Ocean lineage. A similar pattern was also found in the population from Japan (JIM), and its haplotypes clustered with the SCS cluster at three of the seven loci, but with the Indian Ocean cluster at the remaining four loci.

As described in the Introduction, 12 of 71 genes were highly divergent (F_ST_ = 1) between the Qionghai and Sanya populations, although they are separated by only 100 km and are connected by ocean currents [31]. This pattern has been proposed to be local adaptation, which counteracts the homogenizing effect of gene flow between the two populations. In this study, our results suggested that the source of adaptation in the two Hainan populations came from standing genetic variation. In *S. alba*, genetic divergence between the SCS and the Indian Ocean populations could accumulate in isolation stages, and interglacial gene flow could drive the homogenization of most genomic regions, except for those under selection. Some genetic variation may be neutral in one environment but adaptive in new habitats. This fitness advantages can drive these alleles to a high frequency over a short period of time, and can even reach fixation within a population. Moreover, local adaptation could also counteract the immigration of external alleles from other SCS populations, as suggested by Zhou et al. [31].

In general, populations that evolve in long-term isolation have been assumed to be adapted to local conditions and may not be suited to new habitats [60]. However, our study suggests that mangroves can reuse standing genetic variation to adapt to new habitats. Adaptation by use of standing variations has also been observed in other marine species, such as threespine sticklebacks [61].

## Conclusion

Mangrove communities were highly impacted by Pleistocene sea-level fluctuations. In the present study, we propose that genetically distinct populations of *S. alba* from the SCS, the Indian Ocean and Australasia should be considered as three management units (MUs). Furthermore, our results exhibited convincing evidence for interregional genetic exchanges during interglacial stages and addressed adaptive advance of standing genetic variations in facilitating mangrove establishment. These findings provide new insights into the conservation of mangrove plants and for future restoration efforts.

## Methods

### Plant materials

A total of 388 individuals from 22 populations of *S. alba* were sampled across the IWP region. In the IWP region, there are two centres of mangrove biodiversity: Indo-Malesia (from India to Southeast Asia) and Australasia (from North Australia and New Guinea to islands in the western Pacific) [62]. Within the Indo-Malesia region, 15 populations were sampled, including two populations from China, four from Thailand, four from Malaysia, three from Indonesia, two from Philippine and one from Kenya. In the Australasia region, two populations were sampled from Australia, and one from Papua New Guinea. Detailed information regarding sampling location and size is provided in Table 1. For each population, one leaf was collected from each of 10 to 25 individuals and stored in a plastic bag with silica gel for subsequent DNA extraction.

**Table 2:** Maximum-likelihood estimations (MLEs) and 95% of the highest posterior density (HPD) for population parameters of the populations from the SCS (TTN), the Indian Ocean (TPK) and the Indo-Pacific boundary (MKJ) under the estimated substitution rate of 1.616 * 10^−9^ substitutions per site per year (s/s/y; 95% HPD: 1.19–2.09 * 10^−9^ s/s/y) using the isolation with migration model. For each parameter, 95% HPD was shown in parentheses

Parameters	Mutation rate (*10^−9^ s/s/y)		
	1.190	1.616	2.094
t_1_ (MYA)	0.067 (0.000–9.222)	0.049 (0.000–6.791)	0.038 (0.000–5.241)
Ne _TTN_	60 (0–1,015)	44 (0–748)	34 (0–577)
Ne _MKJ_	2,927 (896–9,497)	2,155 (660–6993)	1,663 (509–5397)
Ne _TPK_	537 (179–1,971)	396 (132–1451)	305 (102–1120)
2NM _TTN -> MKJ_	0.727 ^**^(0.089–2.229)		
2NM _MKJ -> TTN_	5.625 *10^−4^ (0.000–0.048)		
2NM _MKJ -> TPK_		0.142 ^**^(0.043–0.347)	
2NM _TPK -> MKJ_		0.002 (0.000–0.603)	
2NM _TTN -> TPK_		0.013 (0.000–0.197)	
2NM _TPK -> TTN_		6.375 *10^−4^ (0.000–0.037)	

“**” denoted *p*-value < 0.01 for migration rate likelihood ratio test, which indicated the migration rate was significantly greater than 0. MYA is short for million years ago

### DNA extraction, PCR amplification and sequencing

Genomic DNA of each individual was extracted using the CTAB method [63]. Three cpDNA fragments and seven nuclear loci were employed to investigate the phylogeography of *S. alba* in this study. *trn*V - *trn*M, *trn*L - *trn*F and matK were amplified for 10 individuals from each population using the corresponding primer pairs (Additional file 6) [64–66], except one population from Cilacap, Java, Indonesia, which has only six samples. Primers of the seven nuclear loci (*rpl9*, *cpi*, *ppi*, *phi*, *cci*, *idr* and *nhx2*) were designed based on the sequences of expression sequence tags (ESTs) from a leaf cDNA library of *S. caseolaris* [67]. Both cpDNA and nuclear loci were amplified by polymerase chain reaction (PCR) using a 30-uL reaction system of KOD FX DNA polymerase (Toyobo Co., Ltd., Osaka, Japan) following the manufacturer’s protocol consisting of an initial melting step at 94 °C for 4 min, 29 cycles of 94 °C for 40 s, 51 °C for 45 s, and 72 °C for 1.25 min, and a final elongation step at 72 °C for 8 min. All PCR products were purified by 2% agarose gel electrophoresis followed by using the StarPrep Gel Extraction Kit (GeneStar Biosolutions Co., Ltd., Beijing, China). The purified products were sequenced by the Sanger method with the corresponding primer pairs in an ABI 3730 DNA analyzer with BigDye Terminator Cycle Sequencing Ready Reaction Kit (Applied Biosystems, Foster city, CA, USA).

### Genetic diversity and divergence

All sequences were aligned using Seqman v. 7.10 (DNAstar, London, UK) and consecutive indels were treated as one insertion/deletion event. The sequences of the three cpDNA fragments were concatenated as a single locus for further analysis. For both chloroplast and nuclear loci, haplotypes were inferred using DnaSP package v. 5.10 [68], and haplotype networks were constructed using the median joining algorithm [69] in NETWORK v. 4.6.1.1 software (Fluxus Technology Ltd., Suffolk, UK). For each population, population genetic parameters, including segregating site number (S), haplotype number (H), haplotype diversity (Hd), nucleotide diversity (θ_π_) and DNA polymorphism (θ_W_), were calculated for each locus using DnaSP.

To identify the genetic structure of *S. alba*, 388 individuals were assigned into a putative number of clusters by the Bayesian clustering method using STRUCTURE v. 2.3.3 [70]. The maximum K was set to 10, and for each K, 20 replicates were carried out. Each run was performed at 1*10^6^ Markov chain Monte Carlo (MCMC) with a burn-in of 2*10^5^ under the assumed model of admixture and correlated allele frequencies. The most likely K was determined by the Delta K statistic according to [71] using STRUCTURE HARVESTER [72], and the results were depicted using DISTRUCT v. 1.1 [73]. In parallel, two parameters of population differentiation, F_ST_ (fixation index) and K_XY_ (average number of nucleotide differences between populations), were calculated between each pair of populations using DnaSP. Based on the result of K_XY_, a neighbor-joining (NJ) tree was constructed for all *S. alba* populations to visualize the genetic differentiation among populations using Mega v. 5.0 [74]. Moreover, to bridge the relationship between genetic divergence and geography, putative geographic barriers were identified among the 22 IWP populations using pairwise F_ST_ values according to Monmonier’s algorithm in Barrier v. 2.2 [75].

### Demographic history inference

Divergence time among different genetic lineages of *S. alba* was estimated using BEAST v. 1.8.2 [76] with four other *Sonneratia* species - *S. caseolaris*, *S. apetala*, *S. ovata* and *S. griffithii* - as outgroups. The topology of the tree prior among the five *Sonneratia* species was set based on the phylogenetic relationship inferred in our previous study [77], while the intra-species relationship of the six *S. alba* populations (including TTN, MSD, PDV, ADW and ADT) was constructed according to the NJ tree for the 22 *S. alba* populations. Due to the lack of an accurate nucleotide substitution rate for *Sonneratia* species, we used the estimated divergence time between *S. ovata*, *S. alba* and *S. caseolaris* as three calibration points under a normal distribution prior with a mean of 7.0, 9.0 and 11.0 million years ago (MYA), respectively, and a standard deviation of 2.0, according to the genomic analysis of mangrove species (He et al. unpublished data).

Furthermore, gene flow between populations from the two sides of the Malay Peninsula was estimated under an isolation with migration (IM) model using IMa2 software [78]. Three populations - TTN, TPK and MKJ - were selected as representatives of populations from the SCS, the Indian Ocean and the Indo-Pacific boundary, respectively (Fig. 5a). For each of the seven nuclear loci, the largest recombination-filtered subset was extracted from the aligned sequences using the perl script IMgc [79] to eliminate the biases of four-gamete violations on demographic history reconstruction. In IM analysis, the HKY substitution model was employed for each locus, with a substitution rate of 1.616 *10^−9^ substitutions per site per year (s/s/y; 95% of the highest posterior density (HPD): 1.190–2.094*10^−9^ s/s/y) estimated by BEAST. According to the posterior distribution of preliminary simulations, the upper ranges for the parameter prior of population size (q), migration rate (m) and divergence time (t) were set at 10, 10 and 20, respectively. To confirm convergence, the simulation was repeated three times with different random number seeds. Each run was implemented at a 2*10^6^ step burn-in under a geometric heating scheme (-hfg -ha0.96 -hb0.9), followed by an MCMC of 1*10^7^ steps. Population parameters, including effective population size (Ne), inter-population migration rate (2NM) and population divergence time (t), were estimated by plotting the marginal posterior probability distributions of 100,000 genealogies, and the optimal estimations were obtained at the peaks of the posterior distribution curves. 95% HPD intervals were also estimated for each parameter, and the likelihood ratio test (LRT) [80] was employed to test the significance of the migration rate.

## Additional files

Additional file 1: Statistics of genetic diversity of the combined chloroplast DNA (cpDNA) and seven nuclear loci in *Sonneratia alba* at both population and species level. Length of each locus was showed in the parentheses after locus ID. Population abbreviations were defined in Table 1. (XLSX 45 kb)
Additional file 2: A heatmap of DNA polymorphism (θ_W_) of 22 populations of *Sonneratia alba*. The color depth and the height of the cylinder are proportional to the level of θ_W_. Population abbreviations were defined in Table 1. (PDF 417 kb)
Additional file 3: Geographic distribution of haplotypes and Median-Joining network for five nuclear loci (a: ppi, b: phi, c: cci; d: idr and e: nhx2) in 22 populations of Sonneratia alba. Each haplotype was represented by one single circle and haplotype frequency was illustrated by circle size. Haplotypes with close relationship were denoted by the same color. The number of mutations is 1 unless otherwise indicated. Population abbreviations were defined in Table 1. (TIF 549 kb)
Additional file 4: A diagram for inferring the optimal K obtained by STRUCTURE across the 22 populations of *Sonneratia alba* using ΔK statistic. (PDF 77 kb)
Additional file 5: Neighbor-joining (NJ) tree showing genetic structure among 22 populations of *Sonneratia alba*. Differential clades were denoted by different colors. (PDF 145 kb)
Additional file 6: Gene IDs, gene descriptions, primer sequences and GenBank accession numbers of haplotypes for the chloroplast DNA (cpDNA) fragments and nuclear loci used in this study. (XLSX 35 kb)
