# Supplementary figures and images for: Effects of Pleistocene sea-level fluctuations on mangrove population dynamics: a lesson from Sonneratia alba
Source: BMC Evol Biol. 2017 Jan 18;17:22. doi: 10.1186/s12862-016-0849-z (PMC5241957; doi:10.1186/s12862-016-0849-z)

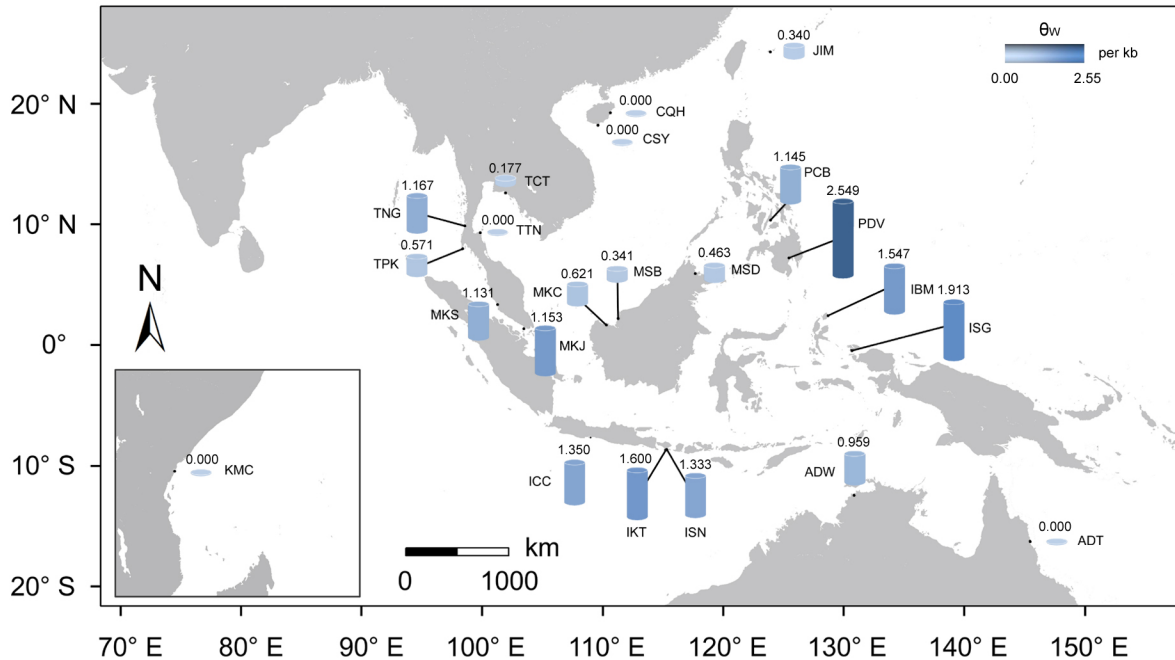

Supplement: Additional file 2: — A heatmap of DNA polymorphism (θW) of 22 populations of Sonneratia alba. The color depth and the height of the cylinder are proportional to the level of θW. Population abbreviations were defined in Table 1. (PDF 417 kb) [file 12862_2016_849_MOESM2_ESM.pdf]

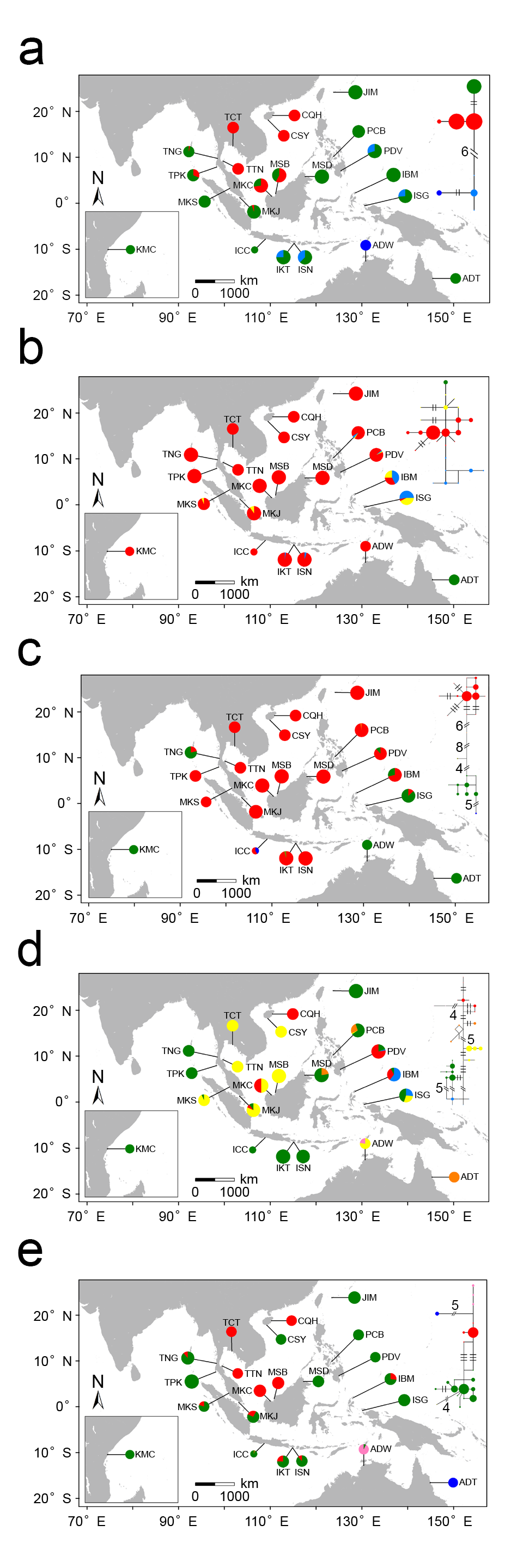

Supplement: Additional file 3: — Geographic distribution of haplotypes and Median-Joining network for five nuclear loci (a: ppi, b: phi, c: cci; d: idr and e: nhx2) in 22 populations of Sonneratia alba. Each haplotype was represented by one single circle and haplotype frequency was illustrated by circle size. Haplotypes with close relationship were denoted by the same color. The number of mutations is 1 unless otherwise indicated. Population abbreviations were defined in Table 1. (TIF 549 kb) [file 12862_2016_849_MOESM3_ESM.tif]

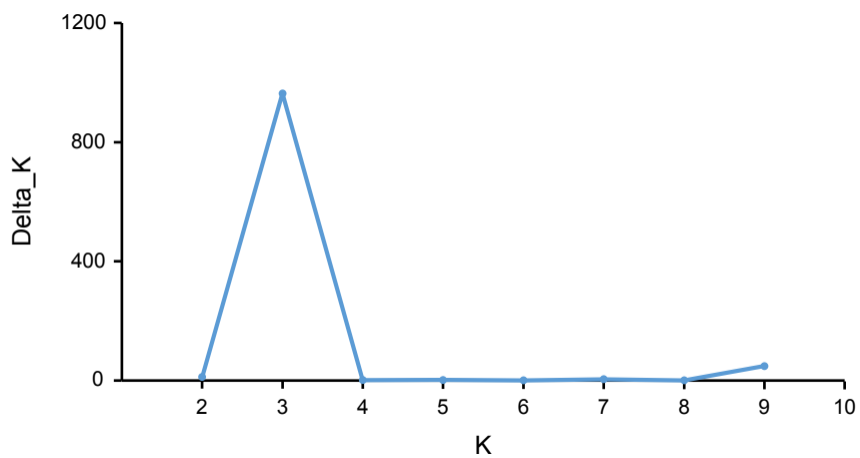

Supplement: Additional file 4: — A diagram for inferring the optimal K obtained by STRUCTURE across the 22 populations of Sonneratia alba using ΔK statistic. (PDF 77 kb) [file 12862_2016_849_MOESM4_ESM.pdf]

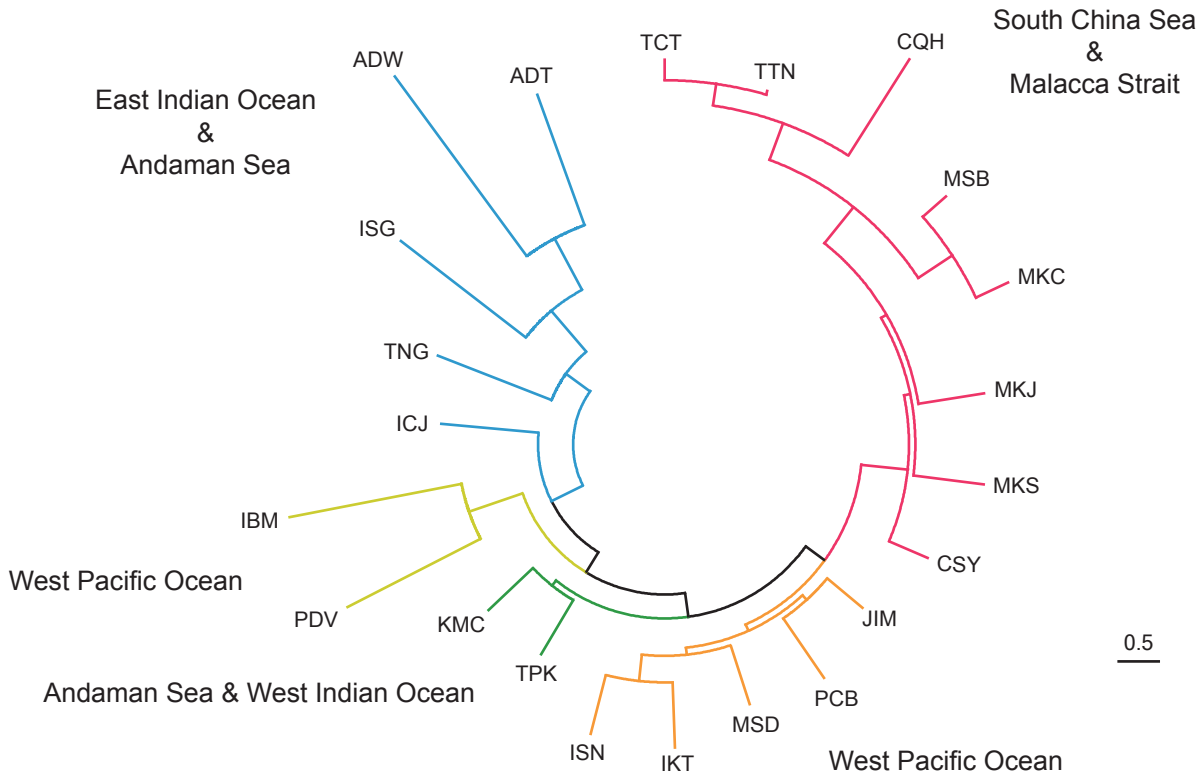

Supplement: Additional file 5: — Neighbor-joining (NJ) tree showing genetic structure among 22 populations of Sonneratia alba. Differential clades were denoted by different colors. (PDF 145 kb) [file 12862_2016_849_MOESM5_ESM.pdf]
